# Detection and activity of MMP-2 and MMP-9 in *Leishmania amazonensis* and *Leishmania braziliensis* promastigotes

**DOI:** 10.1186/s12866-023-02973-z

**Published:** 2023-08-16

**Authors:** Brenda Furtado Costa, Tarcísio Navegante de Queiroz Filho, Adeniele Lopes da Cruz Carneiro, Aline Semblano Carreira Falcão, Maria Sueli da Silva Kataoka, João de Jesus Viana Pinheiro, Ana Paula Drummond Rodrigues

**Affiliations:** 1 Laboratory of Electron Microscopy, Section of Hepatology, Evandro Chagas Institute, Belém, Pará Brazil; 2 Department of Oral Pathology, School of Dentistry, Federal University of Para, Belém, Pará Brazil

**Keywords:** Leishmaniasis, MMP-2, MMP-9, Collagen matrix

## Abstract

Metalloproteinases (MMPs) are remarkable zinc-dependent endopeptidases, critical for degrading components of the extracellular matrix, thus actively influencing cell migration. Their impact on intracellular parasites, such as the enigmatic protozoan Leishmania, elicits intriguing queries. This study explores into the untapped territory of MMP-2 and MMP-9 within Leishmania spp. promastigotes. Notably, we successfully detected and quantified these MMPs, while also evaluating their activity in two distinct Leishmania species—*L. amazonensis* (La) and *L. braziliensis* (Lb)—at various growth stages and isolated from distinct clinical tegumentar disease forms. The results unveiled the presence of MMP-2 and MMP-9 in both species, albeit with distinct localization patterns. Specifically, MMP-9 exhibited significantly higher gelatinolytic activity in La when compared to Lb. Moreover, our data cleverly illustrated the presence and release of MMP-2 and MMP-9 by La and Lb promastigotes, exposing their ability to invade and migrate within a collagen matrix. This pioneering study establishes a compelling correlation between MMP-2 and MMP-9 and their potential role in the dynamics of La and Lb infection. Suggesting their potential as prognostic markers for severe leishmaniasis and promising target molecules for therapeutic interventions, this research opens new avenues for combatting this debilitating parasitic disease.

**Supplementary Information:**

The online version contains supplementary material available at 10.1186/s12866-023-02973-z.

## Introduction

The *Leishmania* protozoan, an obligate intracellular parasite, is the causative agent of leishmaniasis and its target cells are macrophages. Leishmaniasis is a zoonosis that comprises a spectrum of skin and gastrointestinal diseases. Leishmaniasis has been recorded in over 98 countries in the world, and mainly occurs in tropical countries. In the Americas, 39,705 new cases of CL and ML were recorded in 2020. In the same year, Brazil was among the top five countries with highest number of reported cases, with 16,432 new registered cases (SisLeish—OPS/OMS).

In general, pathogens need several molecules for a successful infection, including matrix metalloproteinases (MMPs), which are mainly involved in extracellular matrix (ECM) degradation. At the time of the blood meal, the vector inserts the parasite into the skin of the vertebrate host, and once at the site of the injury, the promastigote migrates through the damaged ECM. The parasite interacts with the molecules present in this microregion, such as fibronectin, laminin and collagen I, until the arrival of defense cells. MMPs are Zn^+ 2^-dependent enzymes that degrade ECM proteins and are classified according to their structure and substrate. MMP-2 and MMP-9 are gelatinases that degrade collagen, fibronectin and plasminogen [1, 2].

Some studies have reported an increase in gelatinase levels during *Mycobacterium tuberculosis*, protozoan *Leishmania sp.* and *Trypanosoma cruzi* infections in vitro [3–6]. However, other studies have detected the presence of the Zn^+ 2^-dependent proteins in the protozoan themselves.

Interestingly, it was reported that a 40 kDa protein in *Trypanosoma brucei brucei* presented gelatinolytic activity, which was inhibited by the administration of EDTA, EGTA, tetracycline, doxycycline and phenanthroline—MMP inhibitors—suggesting the presence of MMP in this trypanosomatid [7]. In the same year, the presence of an MMP-9-like protein in *T. cruzi* was described, and it is similar to the mammalian MMP-9 protein [8]. Cuevas et al., 2003[9] reported the presence of two molecules in *T. cruzi*, Tcgp63-I and Tcgp63-II that were homologous to glycoprotein 63 (gp63), an MMP present in Leishmania. The well-known Leishmania parasite metalloproteinase gp63 belongs to a large family of Zn^+ 2^-dependent proteins called metzincins [10]. Gp63 was discovered in 1980 and described as the major surface antigen of promastigotes. It is an important modulator of the immune response in favor of the parasite [11, 12]. In promastigotes, gp63 inactivates the complement system factor C3b during major infections caused by *L. amazonensis* (La) and *L. braziliensis* (Lb) [12]. Furthermore, the presence of this protozoan MMP has an important role in modulating the mammalian host immune response to promote successful infection. After parasite phagocytosis, amastigote gp63 modulates the host cell through cleavage of important molecules such as NF-κB. After NF-κB is cleaved, is upregulates the expression of chemokines, which attracts more phagocytes and, consequently, presents new target cells for intracellular parasite development [13]. Thus, the aim of this study was to investigate two gelatinases, MMP-2 and MMP-9, in the leishmania promastigote species from the Viannia and Leishmania subgenera isolated from different clinical strains of tegumentary leishmaniasis.

## Materials and methods

### Parasites

Promastigote forms of *Leishmania (Leishmania) amazonensis* (MHOM/BR/26,361) (La) and *Leishmania (Viannia) braziliensis* (MHOM/BR/M17323) (Lb) were obtained in Novy-MacNeal-Nicolle (NNN) medium from the Leishmaniasis Program of the Evandro Chagas Institute and maintained in Roswell Park Memorial Institute (RPMI 1640) medium supplemented with 10% fetal bovine serum (FBS) at 27 °C, according to [14]. Parasites were maintained in culture and were used in this study once they were in the following growth phases: two days of growth (logarithmic phase—LOG), seven days of growth (stationary phase—STAT) and nine days of growth (late stationary phase—LSTAT). *Leishmania* promastigotes were treated with doxycycline (DOX) at a concentration of 50 µM/mL [15, 16] for 24 h at 27 °C according to [8].

### Detection and localization of promastigote gelatinases

#### Flow cytometry and confocal microscopy

Leishmania promastigotes were fixed with 3% formaldehyde, permeabilized with 0.3% Triton X-100, blocked with 50 mM NH_4_Cl and 10% goat serum solution after incubation with the primary antibodies rabbit polyclonal anti-MMP-2 (Novusbio®) or rabbit polyclonal anti-MMP-9 (EMD Millipore®) at 1:100 and incubated overnight in a humid chamber at 4 °C. Subsequently, they were subjected to successive washes with PBS-BSA-TWEEN-20 (phosphate buffer saline, 3% or 1% BSA bovine serum albumin and 0.01% Tween 20). After that, the cells were incubated with goat anti-rabbit secondary antibody conjugated to Alexa® 488 or Alexa® 568 and analyzed using a BD FACS Canto II flow cytometer and DIVA software, and the data obtained were analyzed by Flowing Software (version 2.5.1). Then, the number of positive cells and the fluorescence intensity were analyzed, and the ratio between these two metrics was calculated. This method allowed us to quantify and compare the presence of intracellular MMP.

For fluorescence microscopy, the same protocol described above was used. In addition, the cell nuclei were labeled with 4’,6-diamidino-2-phenylindole (DAPI) and the cells were mounted with Prolong Gold antifade®. The cells were analyzed using a Leica® *TSC SP8 DSL* confocal microscope and LAS X software.

#### Immunolocalization by transmission electron microscopy (TEM)

For immunolocalization of MMP-2 and MMP-9, the promastigotes processing was performed as previously described [17]. The cells were fixed with 0.5% glutaraldehyde, 4% formaldehyde, 3.5% sucrose, 0.1 M sodium cacodylate and 5 mM sodium chloride. After that, the samples were dehydrated in an increasing series of ethanol at low temperature and embedded in LR White hydrophilic resin (Sigma Aldrich®). Ultrafine Sect. (60 μm) were obtained using an ultramicrotome (Leica EM UC6) and collected in gold grids. The grids were incubated with 50 mM NH_4_Cl solution, followed by incubation with PBS (pH 8.0) containing 1% BSA solution and 0.01% Tween 20, and then incubation with 10% goat serum. Subsequently, the samples were incubated with polyclonal anti-MMP-2 primary antibody (Novusbio®) or anti-MMP-9 (EMD Millipore®) at 1:100 overnight in a humid chamber at 4 °C, followed by incubation with goat anti-rabbit secondary antibody conjugated with colloidal gold 15 nm (Electron Microscopy Science©). Sample analysis was performed using an EM 900 Zeiss electron microscope.

### Detection and quantification of promastigote gelatinases in culture supernatant

#### Supernatant culture

Supernatants of both species of *Leishmania* were concentrated with 3k Amicon® Ultracel® Merck Millipore®, and promastigotes were lysed with a solution containing 4% SDS (sodium dodecyl sulfate) for 30 min at 4 °C. Total protein concentration was quantified according to the Bradford-BCA method using a BCA Quantification Protein Kit (Thermo Fisher® or Abcam®) according to the manufacturer’s instructions. Both extract and supernatant were loaded on the gel with 10% SDS ratios of 2:1 and 1:1, respectively, in sample buffer with or without β-mercaptoethanol.

#### Zymography

The promastigote supernatant, with or without DOX, was concentrated with 3 kDa Amicon Ultra® filters (Merck Millipore®) and centrifuged at 10.000 RPM for 5 min at 4 °C. The protein concentration was quantified using a BCA Assay Quantification Protein Kit (Abcam®) according to the manufacturer’s instructions. Then, 10 µg of protein from the promastigote culture supernatant was subjected to electrophoresis in a polyacrylamide gel (10%) containing 0.1% type B bovine gelatin. Then, the gel was washed with 1x Zymogram Renaturating Buffer (Biogen®) and incubated with 1x Zymogram Developing Buffer (Biogen®) at 37 °C overnight. Then, the gel was incubated with a solution containing methanol:acetic acid:distilled water (4.5:1:4.5) and stained with 0.2% Coomassie blue G (Amersham Co®) for 4 h. Finally, the gel was washed with methanol:acetic acid:distilled water for 30 min to remove excess dye. The gel was digitized using an HP scanjet g4050 photo scanner, and the pixel density of the bands was measured using ImageJ® software (version 1.8).

### Promastigote migration in the collagen matrix

For the promastigote migration assay, first, the collagen matrix was prepared according to the laboratory’s internal protocol with the addition of the following solutions at low temperature: PBS (Saline Phosphate Buffer), RPMI 1640 (10X), 1.3 mg/mL collagen (Gibco®) and reconstitution buffer (0.26 M NaHCO2, HEPES 0.2 M). Then, the solution was distributed into the wells of excavated slides and incubated at 37 °C for at least 15 min for complete polymerization.

After polymerization of the matrix, the STAT-Leishmania promastigotes were centrifuged at 1.500 RPM at 26 °C, washed and counted. The cell concentration was adjusted to 1 × 10^6^ cells/mL diluted only in RPMI 1640, and then the promastigotes were added on top of the collagen matrix. Experimental groups were created and then one of two different protease inhibitors was added to the matrix with the promastigotes: Protease Inhibitor Cocktail (AEBSF at 2 mM, Aprotinin at 0.3 µM, Bestatin at 116 µM, E-64 at 14 µM, Leupeptin at 1 µM and EDTA at 1 mM) at a concentration 0.1% [18] named IP and doxycycline at a concentration of 50 µM [15, 16] named DOX. After that, the slides were maintained at 26 °C for different periods of time (30 min and 24 h). After those periods, they were fixed with 3% formaldehyde for 30 min and then washed with PBS. The nucleus was stained with DAPI for 15 min, and then slides were analysed using confocal microscopy. The image acquisition and parameters of the SP8 Leica confocal microscope were determined using the LAS X software to visualize parasite migration in three-dimensional images. The depth and distance traveled by the parasite were determined using numerical rulers and color diagrams generated by the software, which considered the x, y and z axes.

### Statistical analysis

The data were analyzed in GraphPad Prism 7® software using Student’s t test with an alpha level of 95%, and a statistically significant difference was considered when the p value was lower than 0.05 (p < 0.05).

## Results

### Detection of MMP-2 and MMP-9 in promastigotes of *Leishmania* spp. by confocal microscopy

**Fig. 1:**
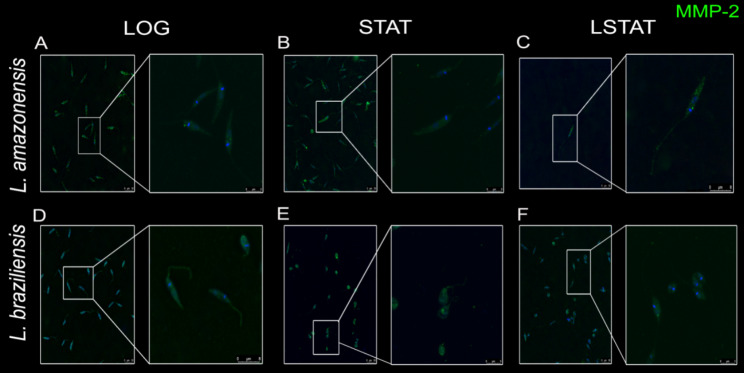
Promastigotes of *Leishmania amazonensis* (**A**, **B**, **C**) and *Leishmania braziliensis* (**D**, **E**, **F**) labeled for detection of MMP-2 at different growth stages. LOG: Logarithmic phase of growth (2 days). STAT: Stationary phase of growth (7 days). LSTAT: Late stationary phase of growth (9 days). Bar: 20 μm. Blue: DAPI. Green: MMP-2

**Fig. 2:**
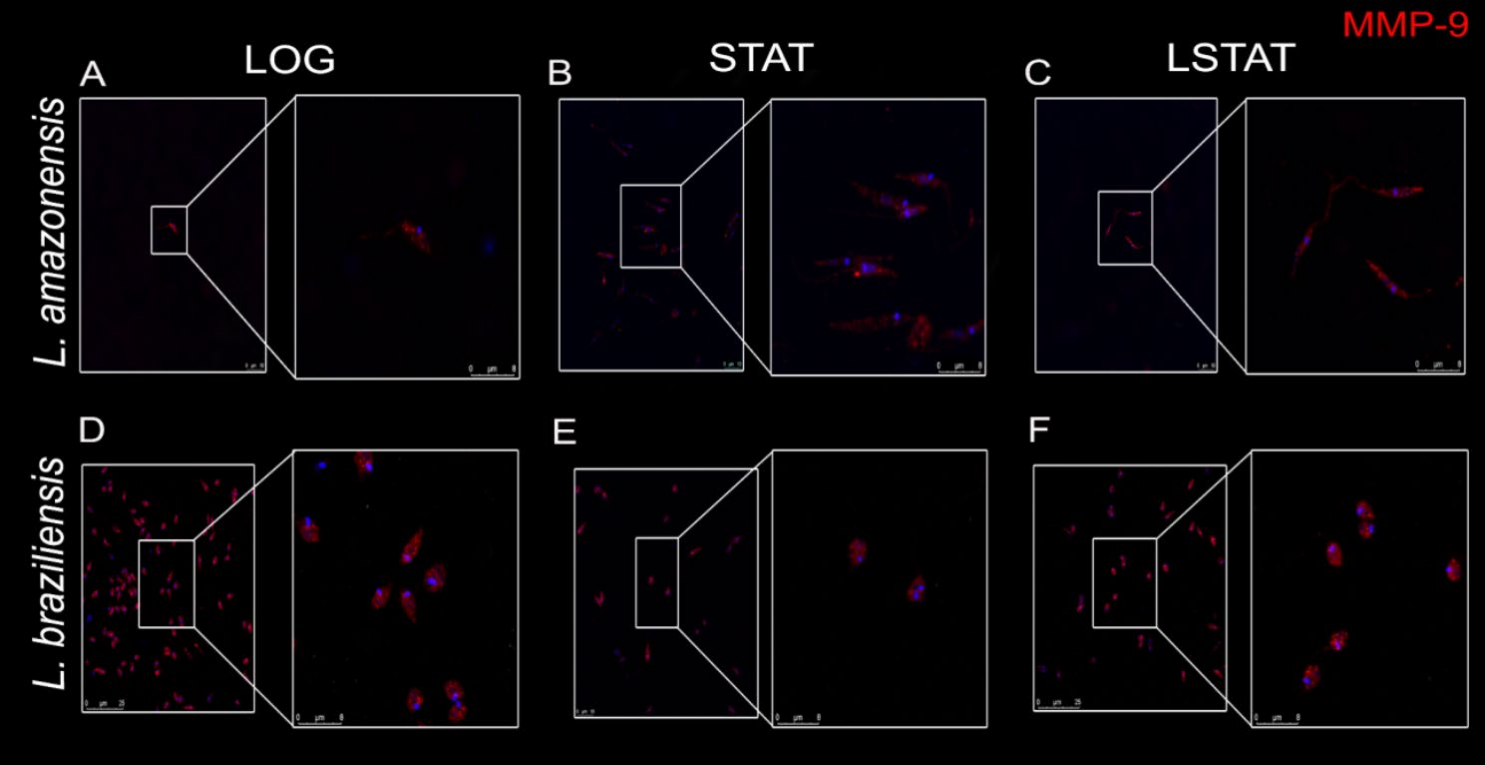
Promastigotes of *Leishmania amazonensis* (**A**, **B**, **C**) and *Leishmania braziliensis* (**D**, **E**, **F**) labeled for detection of MMP-9 at different growth stages. LOG: Logarithmic phase of growth (2 days). STAT: Stationary phase of growth (7 days). LSTAT: late stationary phase of growth (9 days). Bar: 20 μm. Blue: DAPI. Red: MMP-9

MMP-2 was detected in all growth phases for both species. However, for La-LOG, we observed dispersed staining throughout the parasite body (Fig. 1A), while for La-STAT, punctate staining was observed (Fig. 1B), and La-LSTAT exhibited parasite flagellum staining (Fig. 1C). For the Lb parasites, it was not possible to observe distinct MMP-2 distribution, as MMP-2 staining remained dispersed throughout the cellular cytoplasm in all phases (Fig. 1D—F). However, MMP-9 staining proved to be the opposite, where it was not possible to observe distinct distribution in the La parasites. The La parasites presented dispersed labeling through the cytoplasm (Fig. 2A—C), while Lb parasites presented punctual detection in all phases (Fig. 2D—F), mainly in the Lb-LOG parasites (Fig. 2D).

### Localization of MMP-2 and MMP-9 in promastigotes of *Leishmania spp.* by TEM

**Fig. 3:**
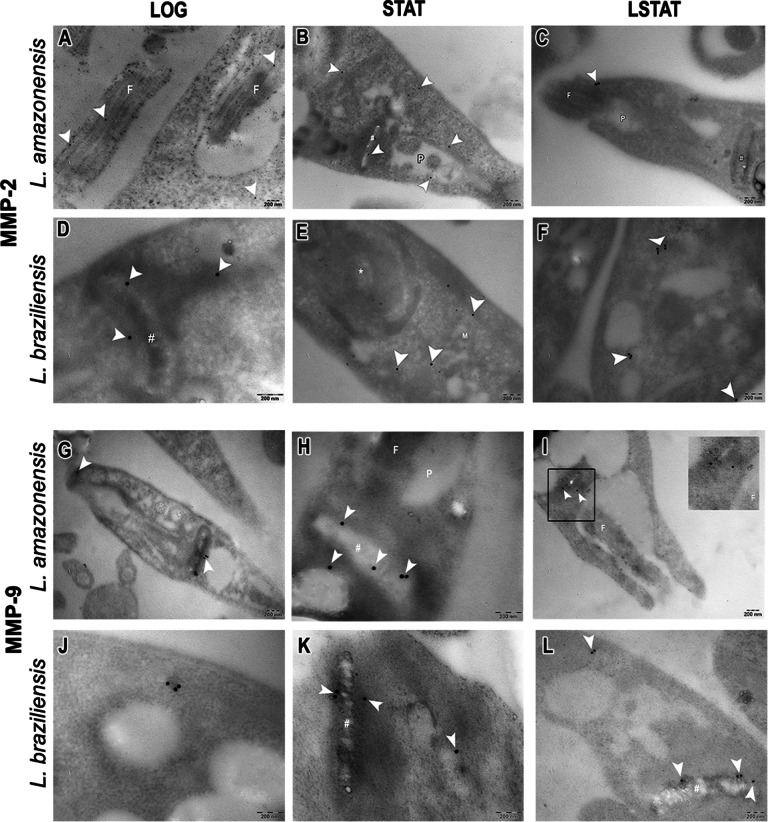
Immunogold labeling for the detection of MMP-2 and MMP-9 in promastigotes of *Leishmania amazonensis* (**A, B, C, G, H and I**) and *Leishmania braziliensis* (**D, E, F, J, K and L**). **#**: kinetoplast. *: nucleus. **M**: mitochondria. **P**: flagellar pocket. **Arrowhead**: 15 nm-colloidal gold. **LOG**: Logarithmic phase of growth (2 days). **STAT**: Stationary phase of growth (7 days). **LSTAT**: late stationary phase of growth (9 days). **Bars**: 200 nm

Through TEM analysis, it was possible to observe the presence and localization of MMP-2 and MMP-9 in both species of *Leishmania* in all analyzed growth phases. In La-LOG, MMP-2 was immunolocalized in the flagellum (Fig. 3A), while in La-STAT, MMP-2 was immunolocalized in the kinetoplast and near the flagellar pocket (Fig. 3B). Finally, in La-LSTAT, MMP-2 was also detected close to the flagellar pocket (Fig. 3C). In Lb-LOG, MMP-2 was observed near the kinetoplast (Fig. 3D). Lb*-*STAT promastigotes still maintained slight detection in the cellular cytoplasm (Fig. 3E). Finally, Lb-LSTAT labeling was dispersed throughout the cell cytoplasm in clusters (Fig. 3F). In contrast, La-LOG showed MMP-9 localization in the parasite body and near the kinetoplast (Fig. 3G). In La-STAT, it was possible to visualize MMP-9 close to the flagellum and near the kinetoplast (Fig. 3H). La-LSTAT showed MMP-9 localization in the cell cytoplasm and close to the flagellum (Fig. 3I). For Lb-LOG, MMP-9 was present in the cell cytoplasm in clusters (Fig. 3J), while in Lb-STAT, MMP-9 was visualized both in the kinetoplast and dispersed in the cell cytoplasm (Fig. 3K). Lb-LSTAT MMP-9 localization was still near the kinetoplast (Fig. 3L).

### High level of MMP-9 in the parasite body of *Leishmania braziliensis*

**Fig. 4:**
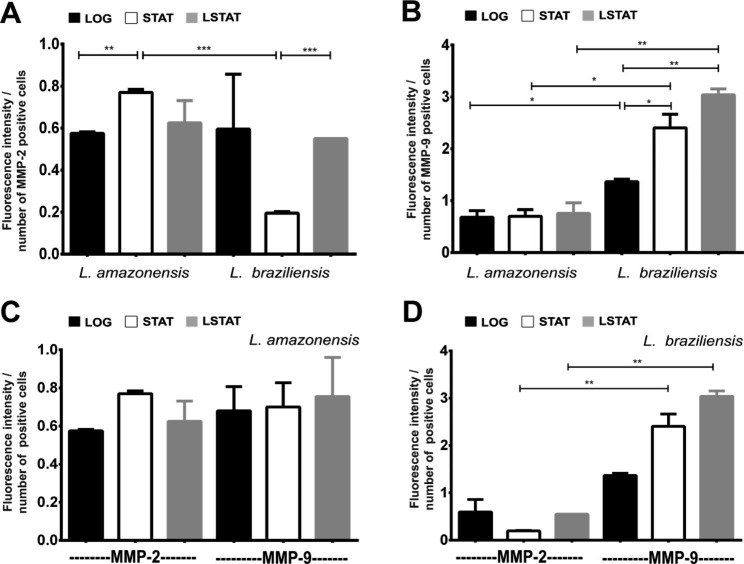
*Leishmania amazonensis* and *Leishmania braziliensis* promastigote detection of MMP-2 **(A)** and MMP-9 **(B)**. Comparison of gelatinase production in *Leishmania amazonensis***(C)** and *Leishmania braziliensis***(D)**. **LOG**: Logarithmic growth phase (2 days). **STAT**: stationary phase of growth (7 days). **LSTAT**: late stationary phase of growth (9 days). *** p < 0.001. ** p < 0.01. * p < 0.05

The promastigotes of La and Lb were immunostained to detect and quantify MMP-2 and MMP-9 in the different growth phases using flow cytometry. Thus, the level of MMP-2 was higher in La-STAT than in La-LOG and Lb-STAT (Fig. 4A). The level of MMP-9 was higher in Lb than in La in all growth phases. Lb-MMP-9 levels increased over time, with higher production in Lb-LSTAT (Fig. 4B). Finally, the production of MMP-2 was compared with the production of MMP-9 for each promastigote species. No differences in the production of the two La-gelatinases (except that previously mentioned) were observed (Fig. 4C). However, for Lb, there was a higher production of MMP-9 in the STAT and LSTAT phases, increasing over time (Fig. 4D).

### MMP-2 and MMP-9 activity in the La and Lb supernatants

**Fig. 5:**
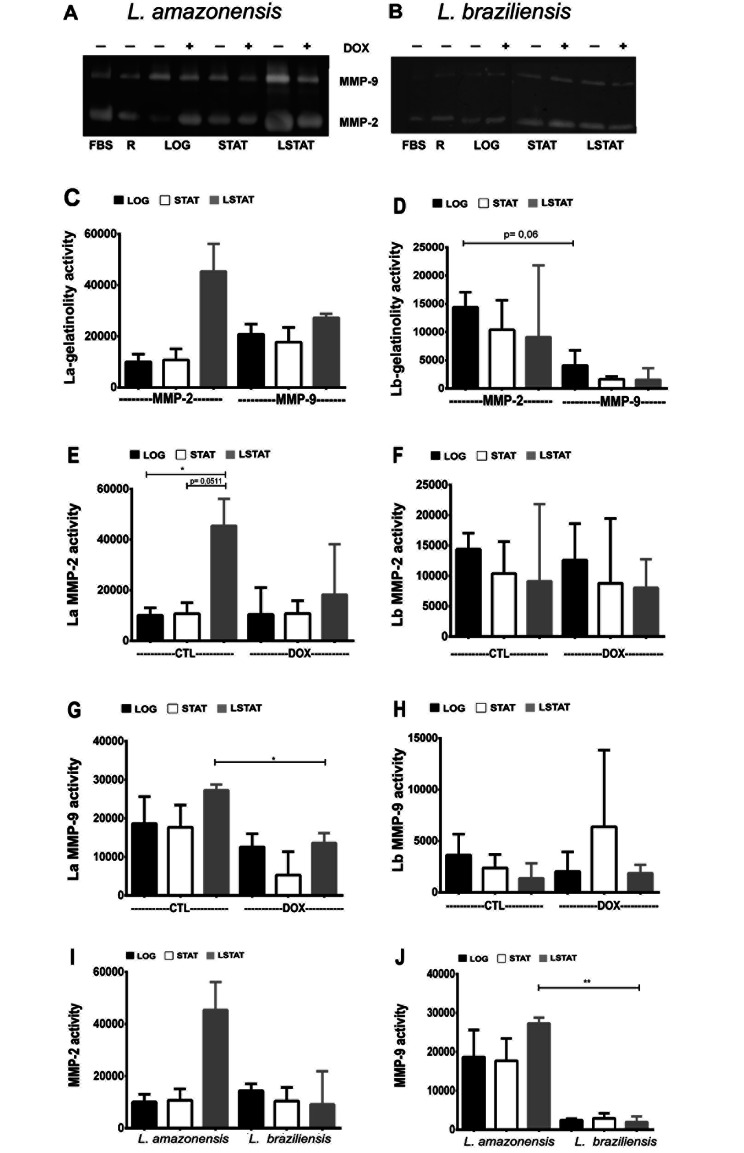
Gelatinolytic activity of metalloproteinases in promastigote supernatant. **(A)** Zymography gel of La. **(B)** Zymography gel of Lb. **(C)** Comparison of gelatinase activity for La. **(D)** Comparison of gelatinase activity for Lb. **(E)** MMP-2 activity of La, with or without DOX. (**F)** MMP-2 activity of Lb, with or without DOX. **(G)** MMP-9 activity of La, with or without DOX. **(H)** MMP-9 activity of Lb, with or without DOX. Optical densitometry comparison between Leishmania species: **(I)** MMP-2 and **(J)** MMP-9. **LOG**: logarithmic growth phase (2 days). **STAT**: stationary phase of growth (7 days). **LSTAT**: late stationary phase of growth (9 days). **FBS**: fetal bovine serum. **R**: RPMI + FBS. **DOX**: doxycycline (50 µM). **p < 0.01, *p < 0.05

After 24 h of MMPs inhibition with DOX, the La and Lb supernatants were subjected to a zymography assay. The gelatinolytic activity of MMP-2 and MMP-9 was observed in all phases and analyzed in the presence or absence of DOX (Fig. 5A and B) for both Leishmania species.

For La, the gelatinolytic activity of MMP-2 and MMP-9 appeared to be higher in LSTAT (Fig. 5C), despite no statistical significance. For Lb-LOG, MMP-2 tended to be more active than MMP-9 in this growth phase (Fig. 5D).

Comparing the inhibited and noninhibited groups, MMP-2 activity was higher in the supernatant of La-LSTAT (Fig. 5E), whereas for Lb, MMP-2 activity was constant in all groups (Fig. 5F). In La, MMP-9 activity was observed in all growth phases, but there was a decrease in MMP-9 activity in the DOX group, demonstrating the effectiveness of MMP-9 inhibition with DOX (Fig. 5G). In Lb, MMP-9 activity showed oscillation without significant loss of activity when inhibited with DOX (Fig. 5H).

Finally, when comparing MMP-2-gelatinolytic activity between species, the La-LSTAT group appeared to have greater activity (Fig. 5I) compared to that of Lb-LSTAT, and this difference was significant when comparing MMP- 9 activity, which was higher in La-LSTAT than in Lb-LSTAT (Fig. 5J).

### *Leishmania spp.* promastigotes migration in collagen matrix

**Fig. 6:**
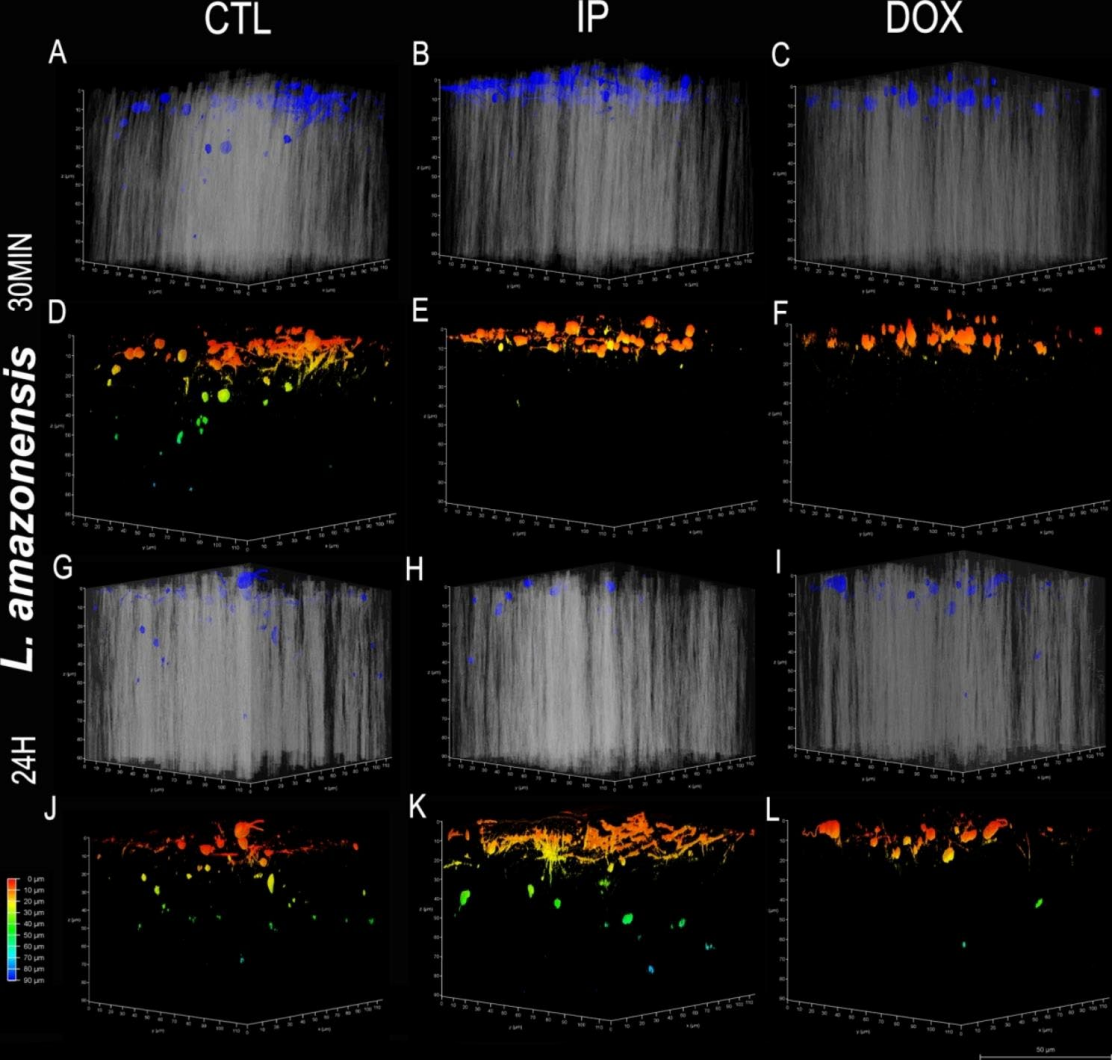
*Leishmania amazonensis* migration assay at different time intervals 30 min (A-F) and 24 h (G-L) and with doxycycline and protease inhibitor cocktail or not. Top images (blue: DAPI and gray: collagen matrix visualized by interference contrast). The color diagram represents the depth (red: 0 μm to blue: 90 μm). **Bar**: 20 μm. **CTL**: control untreated promastigotes; **IP**: promastigotes treated with protease inhibitor cocktail; **DOX**: promastigotes treated with doxycycline

**Fig. 7:**
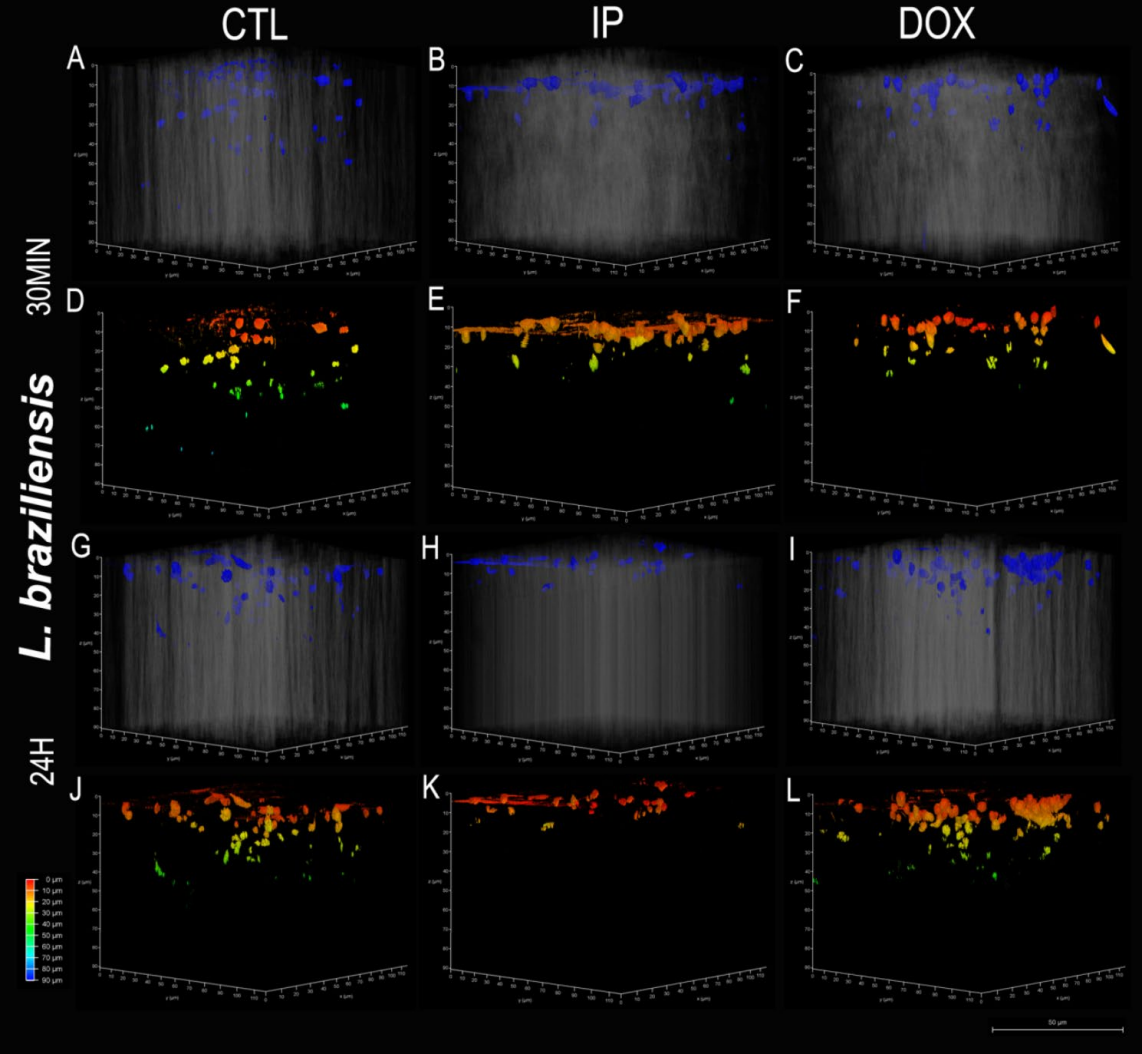
*Leishmania braziliensis* migration assay at different time intervals 30 min (A-F) and 24 h (G-L) and with doxycycline and protease inhibitor cocktail or not. Top images (blue: DAPI and gray: collagen matrix visualized by interference contrast). The color diagram represents the depth (red: 0 μm to blue: 90 μm). **Bar**: 20 μm. **CTL**: control untreated promastigotes; **IP**: promastigotes treated with protease inhibitor cocktail; **DOX**: promastigotes treated with doxycycline

Leishmania promastigotes were added to an already polymerized collagen matrix, with or without inhibitors, and kept at 26 °C for 30 min or 24 h. Collagen invasion and migration of both were observed using confocal microscopy. Visualization was performed to a maximum depth of 90 μm (dark blue) in the matrix for all groups. It is possible to visualize the migration and invasion of Leishmania in the matrix in the figures (Figs. 6 and 7).

**Fig. 8:**
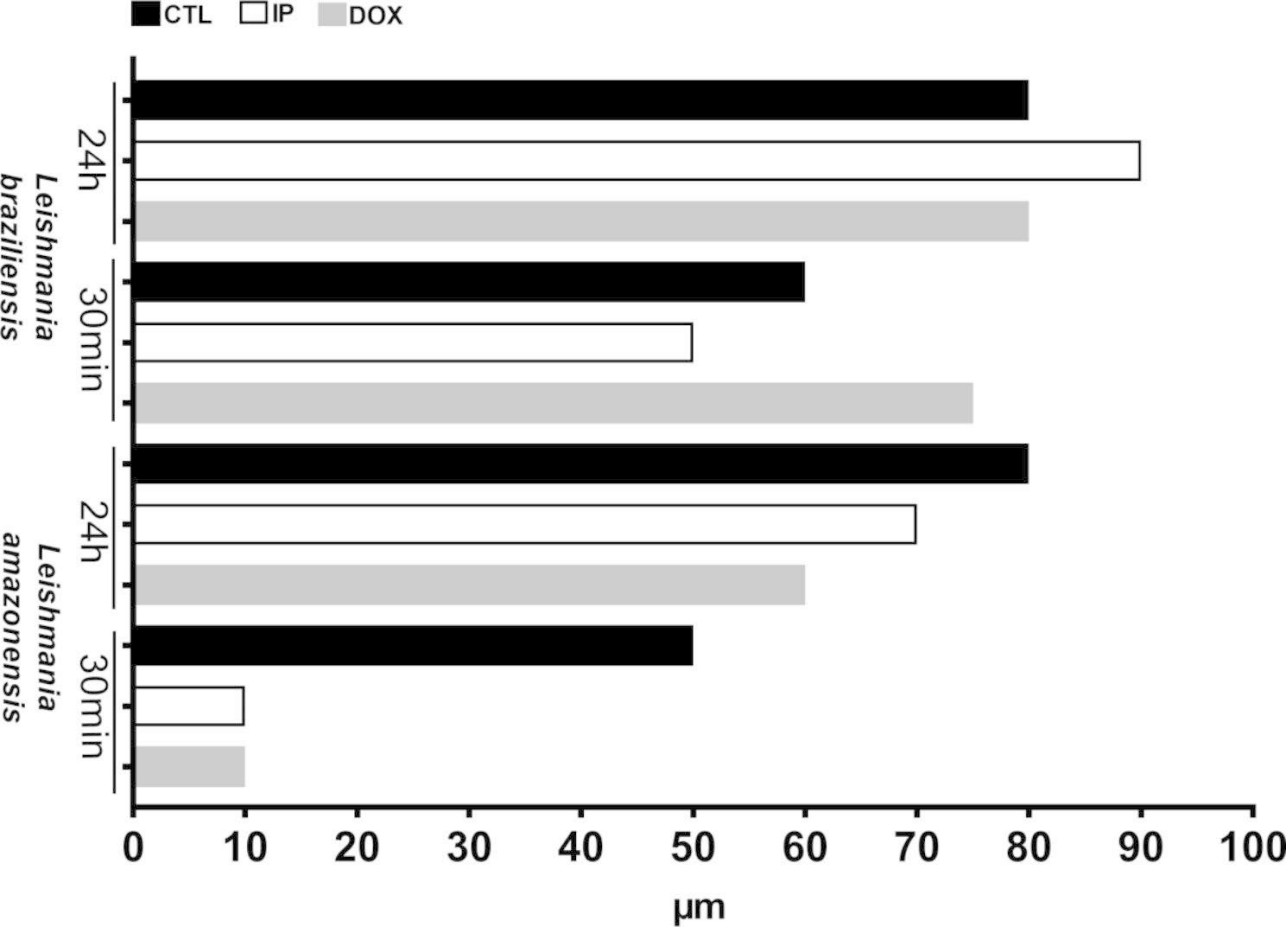
Migration timeline graphic representation of *Leishmania amazonensis* and *Leishmania braziliensis* in the collagen matrix. **CTL La**: La untreated, **IP La**: La treated with protease inhibitor cocktail; **DOX La**: La treated with doxycycline; **CTL Lb**: Lb untreated, **IP Lb**: Lb treated with protease inhibitor cocktail; **DOX Lb**: Lb treated with doxycycline

La promastigote migration was shown to be inhibited both by DOX and IP treatment within 30 min. The control group promastigotes migrated up to 60 μm, and the migration of the inhibitor groups stagnated at 10 μm (Fig. 6D, E, F and Fig. 8). However, after 24 h, the control and IP groups presented similar migration (60 and 70 μm, respectively), but the migration of DOX group was slower or even inhibited, reaching 80 μm (Fig. 6J, K, L and Fig. 8). Lb promastigotes seemed to migrate faster because within 30 min, the control group reached a depth of 60 μm, seen in green in Fig. 7D, while the inhibitor groups reached a maximum average depth of 62.5 μm (Fig. 7E, F), demonstrating migration inhibition. However, 24 h after migration, it was possible to visualize migration in all groups (Fig. 7J, K, L).

Numerically, we visualized the migration of each *Leishmania* in each phase, with inhibitor treatment or not. We identified the last parasite present within each photographed matrix and thus created a migration graph for both species (Fig. 8).

## Discussion

The protozoan Leishmania needs several essential molecules for the successful infection of both vertebrate and invertebrate hosts. At the time of the blood meal, the vector inserts the parasite into the skin of the vertebrate host. Once at the site of injury, the promastigote migrates through the damaged ECM and interacts with the molecules present in this microregion, such as fibronectin and collagen I, until reaching the cells of immune defense [19]. One of the molecules necessary for this migration process is metalloproteinases, including gelatinases and Zn^+ 2^-dependent endopeptidases, that degrade denatured collagen and other types of collagens [1, 2] that make up the ECM.

In this study, the presence of MMP-2 e MMP-9 in the protozoan body of Leishmania in different subgenera (Viannia and Leishmania) was demonstrated. MMP-2 and MMP-9 were observed in the parasite body, including the flagellum, of both *Leishmania braziliensis* and *Leishmania amazonensis*. The culture supernatant analysis suggested the parasite produced, released and activated these molecules, suggesting that these proteins help the parasite in the migration process within the vertebrate host, which was confirmed when we evaluated invasion and migration in the collagen matrix experiments.

First, the promastigotes of La and Lb were analyzed in their three growth phases (logarithmic phase, stationary phase, late stationary phase), and both MMP-2 and MMP-9 were detected using confocal microscopy and TEM. For La, they presented both gelatinases close to the flagellar pocket, which suggests the release of these molecules through this organelle [20], since it is a known area responsible for parasite metabolism and intra and extracellular communication. In addition, these localization data corroborated the greater activity of MMP-2 and MMP-9 observed in the supernatant of the La promastigotes.

For Lb promastigotes, both gelatinases were scattered throughout the cytoplasm and close to the kinetoplast; thus, other molecules may be involved in the migration of this parasite. The distribution and activity variation diversity in the MMPs between these two parasite species investigated in this study highlights the clinical differences of the parasites species. La acts in a balanced way, hides from the immune system, and may cause diffuse cutaneous leishmaniasis, while Lb has a more aggressive character and tropism for the mucous membranes, causing mucocutaneous leishmaniasis.

Investigating the role of MMP-2 and MMP-9 in La and Lb parasites reveals intriguing differences in their localization and production. In the case of La infection, both MMP-2 and MMP-9 seem to be actively involved, showing no statistically significant distinctions between the two species. However, in Lb infection, MMP-9 appears to play a more prominent role than MMP-2. Interestingly, MMP-9 is found in higher levels within the parasite’s body, while their activity levels show no discrepancy between the two gelatinases. This implies that Lb employs MMP-9 in an unidentified manner, suggesting the need for further research.

Gelatinases play an important role in the diagnosis of parasitic diseases, and the presence of high MMP levels is correlated with patient prognosis. Bautista-Lopez et al., 2013[21] found an increase in the activity of MMP-2 and MMP-9 in the plasma of patients diagnosed with Chagas disease and greater MMP-9 activity in patients with abnormal electrocardiograms and cardiomyopathy. In addition, they showed a positive correlation between abnormal cardiac relaxation and elevated levels of MMP-2, demonstrating the possibility of these gelatinases becoming biomarkers in the development of heart diseases in individuals infected with *T. cruzi*. Moreover, Maretti-Mira et al., 2011[5] found gelatinolytic activity in the tissue of patients diagnosed with tegumentary leishmaniasis, which was increased in patients who had a poor response to antimonial treatment. Marangoni et al., 2011 detected the presence of MMP-2 and MMP-9 in the brain spinal fluid (BSF) of dogs naturally infected with *L. chagasi*. Moreover, the presence of MMPs in the BSF seems to cause inflammation in the central nervous system of dogs diagnosed with visceral leishmaniasis, leading to the worsening of the disease in the animals.

In addition to the activity observed by zymography, this study determined that the proteins were between 60 and 160 kDa (S1), corroborating the diverse MMP characteristics and gelatinolytic activity described thus far. Two strains of Lb demonstrated the presence of peptidase. One strain released three enzymes (120, 66 and 45 kDa), while the other released only one 66 kDa enzyme [22]. *Leishmania L. tropica*, *L. major and L. infantum* released different proteins in gels containing gelatin with sizes between 60 kDa and 70 kDa [27]. In addition, an MMP-9-like gelatinase was found in the trypomastigotes of *T. cruzi*, confirming the presence of MMPs in these parasites [8].

The variability observed could be related to the virulence potential of these peptidases; however, these studies did not mention in which growth phase the parasites were evaluated. For the first time, the production of MMPs in Leishmania species was evaluated. In our study, there was variation in the production of these proteins in different phases of parasite growth, mainly STAT, which is the infective phase of parasites [23, 24].Finally, the last objective of this study was to determine whether MMP aids the invasion and migration process of La and Lb promastigotes in the collagen matrix. In the first 30 min, the MMP inhibitors seemed to work, mainly for La promastigotes, but after 24 h, both La and Lb promastigotes could migrate through the collagen matrix, as previously demonstrated [25]. Lb promastigotes were also able to migrate through the at a faster rate than that of La promastigotes, which can be attributed to its aggressive nature, as this species has the ability to migrate to different regions of vector inoculation, such as the mucosal region [26]. A study showed an increase in MMP-9 release by cells from individuals with cutaneous leishmaniasis compared to healthy individuals [3].

This study represents a pioneering effort in identifying and characterizing MMP-2 and MMP-9 in two Leishmania species, each one associated with distinct clinical manifestations of tegumentar leishmaniasis. The presence of these gelatinases was observed at different stages of promastigote development, including two days of growth (logarithmic phase—LOG), seven days of growth (stationary phase—STAT), and nine days of growth (late stationary phase—LSTAT) in an in vitro culture. The detection of MMP-2 and MMP-9 was confirmed through antigen-antibody interaction, demonstrating their structural affinity with commonly employed antibodies. Furthermore, these two gelatinases were achieved using advanced techniques, including confocal microscopy, flow cytometry, and TEM. This comprehensive dataset surpasses the limitations of previous studies [27].

Remarkably, our study marks the inaugural identification of MMP-2 and MMP-9 in promastigotes forms, satisfying all the specified criteria, including the evaluation of their gelatinolytic activity and molecular weight in kDa. In contrast to our findings, proteins of comparable sizes were observed in axenic amastigotes, suggesting that even in this life form, the parasite releases similar proteins as seen in the promastigote in vitro culture.

## Conclusion

Overall, our data demonstrated for the first time the presence and release of MMP-2 and MMP-9 by promastigotes of *Leishmania amazonensis* and *Leishmania braziliensis*, at different stages of parasite growth, as well as the invasion and migration of promastigotes into a matrix of collagen. This study demonstrated possible proteins used by Leishmania during its development, such as promastigotes that may be necessary for successful infection. In addition, MMPs can be used as prognostic markers of the disease and even target proteins for treatment.

### Electronic supplementary material

Below is the link to the electronic supplementary material.

Supplementary Material 1

## Data Availability

All data generated or analysed during this study are included in this published article.
