# Supplementary material for: Detection and activity of MMP-2 and MMP-9 in Leishmania amazonensis and Leishmania braziliensis promastigotes
Source: BMC Microbiol. 2023 Aug 16;23:223. doi: 10.1186/s12866-023-02973-z (PMC10428646; doi:10.1186/s12866-023-02973-z)
Supplement: Supplementary file 1 — Supplementary Material 1 [file 12866_2023_2973_MOESM1_ESM.docx]

# MATERIALSUPPLEMENTARY

### MATERIALS AND METHODS

### 1.1 SDS‒PAGE ANALYSIS

The promastigotes in the different growth phases were lysed with radioimmunoprecipitation assay buffer (RIPA buffer - Sigma Aldrich®) for 5 min at 4°C and then centrifuged for 10 min at 12.000 RPM. These proteins were quantified, and 7.5 µg of total protein was run on a 10% polyacrylamide-SDS gel for 2 hours. The gel was stained with Coomassie blue-R for 2 hours and digitized to determine the bands.

# 2. RESULTS

### 2.1 Preview protein gel from SDS‒PAGE

The proteins extracted from the promastigote form of Leishmania at different time intervals were lysed, as described above, making it possible to visualize two bands in the gel in the range of 60 kDa, suggesting the presence of gp63 and another unidentified protein of similar molecular weight. (Figure 1).


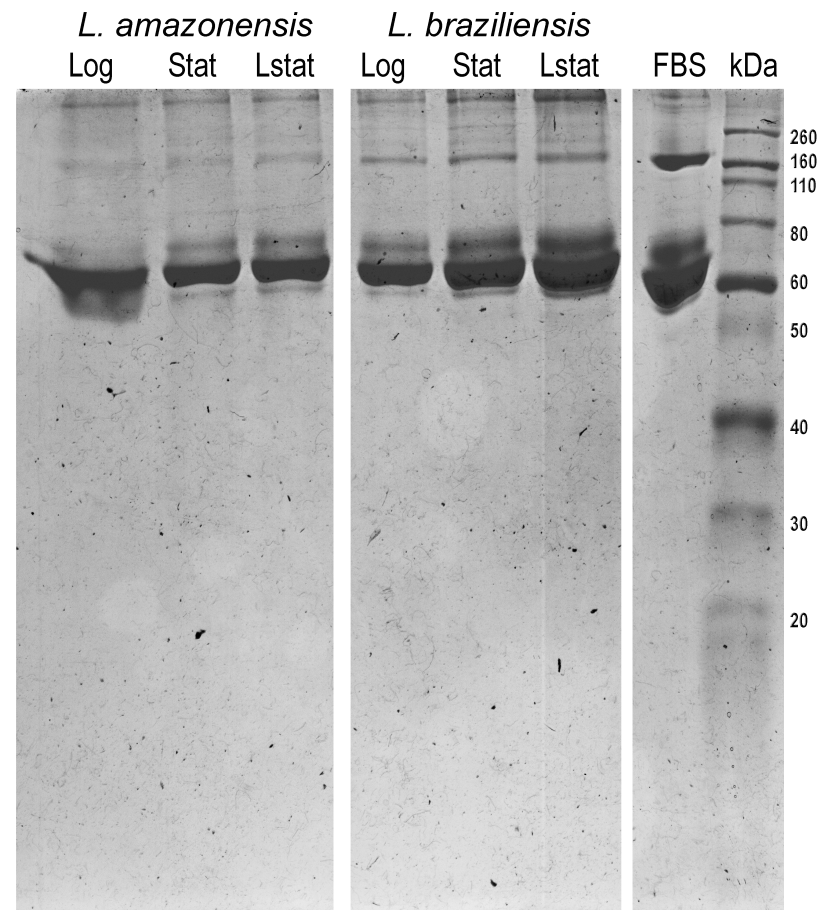


**Figure 1.** SDS‒PAGE gel with 10% polyacrylamide stained with Coomassie blue-R. **FBS:** fetal bovine serum. **LOG:** growth phase (2 days). **STAT:** stationary phase of growth (7 days). **LSTAT:** late stationary phase of growth (9 days).
